# Biological, clinical and epidemiological features of COVID-19, SARS and MERS and AutoDock simulation of ACE2

**DOI:** 10.1186/s40249-020-00691-6

**Published:** 2020-07-20

**Authors:** Xue-Yan Zhang, Hao-Jie Huang, Dong-Lin Zhuang, Moussa Ide Nasser, Ming-Hua Yang, Ping Zhu, Ming-Yi Zhao

**Affiliations:** 1 Department of Pediatrics, The Third Xiangya Hospital, Central South University, Changsha, 410013 Hunan China; 2 Xiangya School of Medicine, Central South University, Changsha, 410013 Hunan China; 3 Guangdong Cardiovascular Institute, Guangdong Provincial People’s Hospital, Guangdong Academy of Medical Sciences, Guangzhou, 510100 Guangdong China

**Keywords:** COVID-19, Coronavirus, SARS, MERS, Autodock

## Abstract

**Background:**

The outbreak of coronavirus disease 2019 (COVID-19) has caused a public catastrophe and global concern. The main symptoms of COVID-19 are fever, cough, myalgia, fatigue and lower respiratory tract infection signs. Almost all populations are susceptible to the virus, and the basic reproduction number (*R*_0_) is 2.8–3.9. The fight against COVID-19 should have two aspects: one is the treatment of infected patients, and the other is the mobilization of the society to avoid the spread of the virus. The treatment of patients includes supportive treatment, antiviral treatment, and oxygen therapy. For patients with severe acute respiratory distress syndrome (ARDS), extracorporeal membrane oxygenation (ECMO) and circulatory support are recommended. Plasma therapy and traditional Chinese medicine have also achieved good outcomes. This review is intended to summarize the research on this new coronavirus, to analyze the similarities and differences between COVID-19 and previous outbreaks of severe acute respiratory syndrome (SARS) and Middle East respiratory syndrome (MERS) and to provide guidance regarding new methods of prevention, diagnosis and clinical treatment based on autodock simulations.

**Methods:**

This review compares the multifaceted characteristics of the three coronaviruses including COVID-19, SARS and MERS. Our researchers take the COVID-19, SARS, and MERS as key words and search literatures in the Pubmed database. We compare them horizontally and vertically which respectively means concluding the individual characteristics of each coronavirus and comparing the similarities and differences between the three coronaviruses.

**Results:**

We searched for studies on each outbreak and their solutions and found that the main biological differences among SARS-CoV-2, SARS-CoV and MERS-CoV are in ORF1a and the sequence of gene spike coding protein-S. We also found that the types and severity of clinical symptoms vary, which means that the diagnosis and nursing measures also require differentiation. In addition to the common route of transmission including airborne transmission, these three viruses have their own unique routes of transmission such as fecal-oral route of transmission COVID-19.

**Conclusions:**

In evolutionary history, these three coronaviruses have some similar biological features as well as some different mutational characteristics. Their receptors and routes of transmission are not all the same, which makes them different in clinical features and treatments. We discovered through the autodock simulations that Met124 plays a key role in the efficiency of drugs targeting ACE2, such as remdesivir, chloroquine, ciclesonide and niclosamide, and may be a potential target in COVID-19.

## Background

The outbreak of coronavirus disease 2019 (COVID-19) caused by a novel coronavirus has become a public health emergency of international concern (PHEIC). The virus can cause severe respiratory disease and can spread from person to person. The virus has been named severe acute respiratory syndrome coronavirus 2 (SARS-CoV-2) based on its appearance under electron microscopy [1]. COVID-19 has been designated a Public Health Emergency of International Concern (PHIEC) [2]. The Chinese Center for Disease Control and Prevention (China CDC) determined that SARS-CoV-2 infection is the cause of the outbreak that started in in Wuhan City (CDC, 2020) [3]. The virus can cause severe respiratory illness, and human to human transmission has been confirmed [4]. Isolated viruses were observed under electron microscopy and named SARS-CoV-2 [5]. As of March 23, 2020, 187 countries and regions reported 348 667 COVID-19 confirmed cases worldwide. Although the COVID-19 situation has been suppressed recently in China, we still need to strengthen the prevention and control of the epidemic, improve people’s awareness of protective measures, and minimize the loss caused by the virus to prevent it from becoming a severe global pandemic.

This is the third time in human history, following SARS and MERS, that a coronavirus has caused a widespread epidemic. Given that the epidemic is still spreading and the evidence that there are similarities among the three coronaviruses in terms of their biological, clinical and epidemiological features, a comparison among the three is very helpful to guide the improvement of treatment and prevention measures, and the similarities and differences among the three are likely to provide the key to addressing the COVID-19 epidemic.

Coronaviruses are large, enveloped, positive-strand RNA viruses that can be divided into four genera: α, β, γ and δ. There are six coronaviruses that cause disease in humans, including 229E and NL63 of the α genus and OC43, HKU1, SARS-CoV and MERS-CoV of the β genus [1, 6–8]. Human coronavirus (hCoV) infection is mainly related to the respiratory, intestinal and nervous systems. In 2002–2003, SARS-CoV caused an epidemic of severe acute respiratory diseases in China; MERS-CoV was found in the Middle East in 2012 [9, 10]. Both of these coronaviruses are zoonotic pathogens that can cause severe respiratory disease in humans, which can progress to severe acute respiratory distress syndrome.

**Fig. 1:**
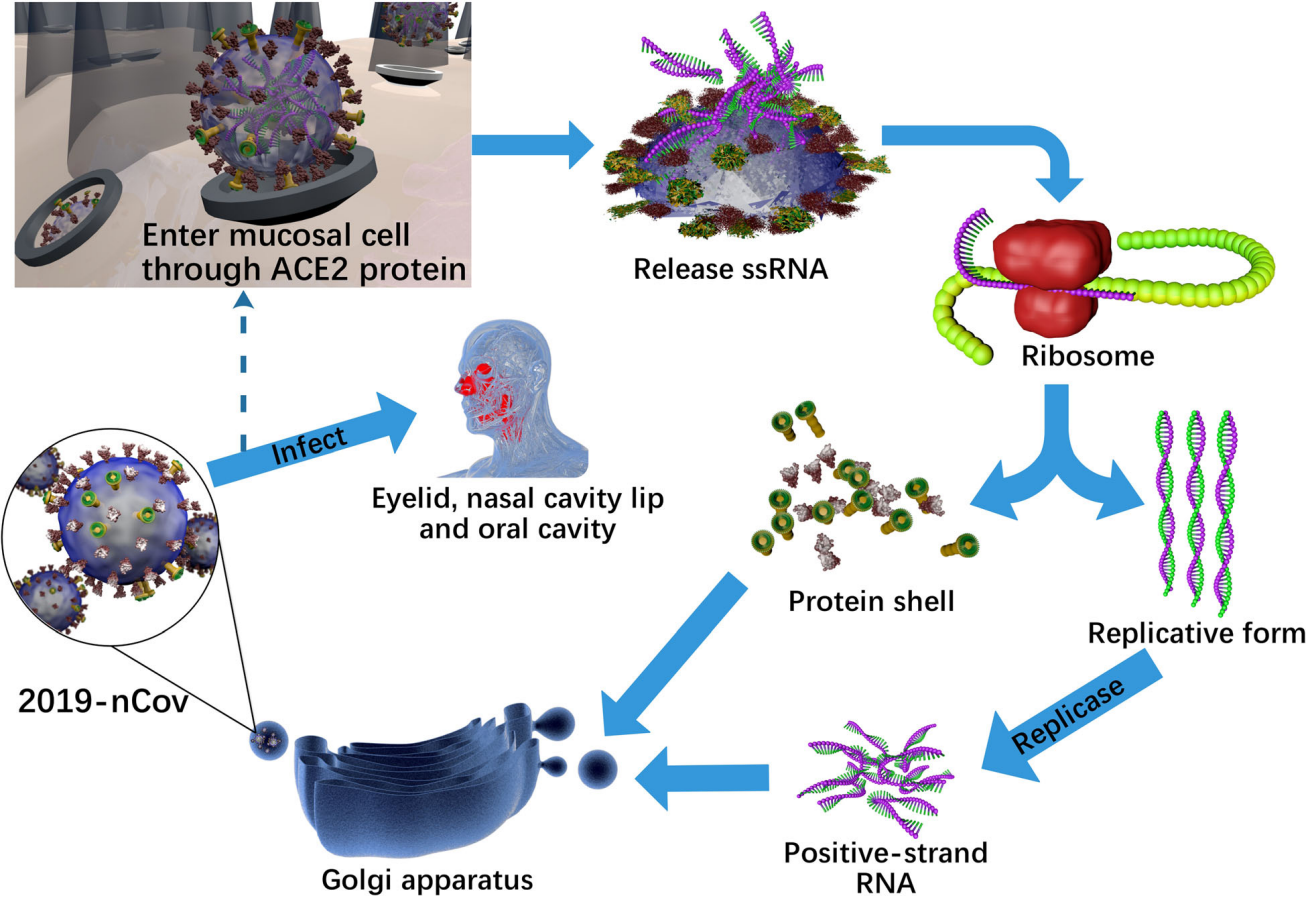
SARS-CoV-2 infects the human body and its replication mechanism. SsRNA: Single string RNA, ACE2: Angiotensin converting enzyme 2

According to the experiment by Peng et al. [5], COVID-19 uses ACE2 as a cellular entry receptor; ACE2 is distributed on the epithelial cells of the alveoli, trachea, bronchi, serous bronchial glands, alveolar monocytes and macrophages in the respiratory tract. ACE2 is also widely expressed on the mucosa, such as the eyelid, nasal cavity, lip, and oral cavity. The pathogenic mechanism of COVID-19 is clear, as shown in Fig. 1. The virus enters the target cells via ACE2 and then releases single-strand RNA (ssRNA), which combines with the ribosome in the target cell and is translated to RNA replicase. The RNA replicas copy the ssRNA to produce negative-strand RNA, positive-strand RNA and RNA fragments, which combine with the ribosome to produce a protein shell. The protein shell and the positive-strand RNA form the new SARS-CoV-2 virions, which are released to infect more target cells (Fig. 1).

This review aims to summarize the biological, clinical and epidemiological characteristics of COVID-19 and to elucidate the current clinical diagnosis and treatment. The comparison of the similarities and differences among the three viruses might be useful for further research on the treatment of COVID-19. Learning from past experience with fighting epidemics, it is very important to understand the epidemiological and clinical characteristics of the virus to control the epidemic, and this review summarizes the existing research and shows a targeted comparative analysis of the characteristics of the three related viruses to provide the scientific basis for later epidemic prevention and control measures. Based on the studies published in the last few weeks, this review shows the characteristics of the spread, diagnosis and treatment of the disease. Recently, many scientists have reported that the pathogenic mechanism of COVID-19 is mainly related to ACE2. The binding affinities of remdesivir, chloroquine, Ciclenosid, Niclosamide and Lopinavirus to ACE2 were determined and compared.

## Methods

We searched the published journal article and website information for COVID-19 in the Pubmed database and Web of science database within one year, including laboratory studies, hospital case reports, and clinical case analysis. We also searched SARS as a key word in the Pubmed database and narrow the range according to the published journal article and governmental policy, speech and report, and read the retrieved literature. The latest version of treatment guidelines for COVID-19, SARS and MERS were also searched in the Pubmed database.

## Main text

### Biological, clinical and epidemiologic features of COVID-19

The comparison of features among COVID-19, SARS-CoV and MERS-CoV is summarized in Table 1.

**Table 1:** Comparisons of the similarities and differences among three coronaviruses

	SARS-CoV-2	SARS-CoV	MERS-CoV
Genus	β-CoVs		
Possible natural reservoir	Bat		
Common symptoms	Fever, cough, myalgia and fatigue		
Susceptible population	All people	All people (a slight female predominance)	All people (predominantly older males)
Respiratory tract symptoms	Pneumonia and lower respiratory signs	Upper respiratory signs and gastrointestinal symptoms	Upper respiratory signs and gastrointestinal symptoms
Receptor	ACE2	ACE2	hDPP4
Intermediary host	Pangolin	Palm civet	Dromedary camel
Origin	Unknown	Guangdong, China	Arabian Peninsula
Route transmission	Airway, contact transmission and aerosol transmission	Respiratory droplets transmission or direct person-to-person transmission through close contact	A combination of long-range airborne and close contact routes
Infected number	5 490 640 (May 24, 2020)	More than 8098	2254 (Sep 19, 2018)
Number of deaths	346 328	916	800
Treatment	No cure, vaccine, or specific treatment	Combination of lopinavir and ritonavir or only ribavirin or IFN-α1 plus corticosteroids	Combination of ribavirin (or ritonavir) and interferon alpha-2b

*ACE2* Angiotensin converting enzyme 2

#### Biological features

With high-throughput sequencing, researchers announced the sequencing of SARS-CoV-2. The genome of SARS-CoV-2 consists of 6 major ORFs that are common to coronaviruses, and the sequence of SARS-CoV-2 has almost 70% similarity to that of SARS-CoV and nearly 40% similarity to that of MERS-CoV [5, 6, 11, 12]. The main differences among SARS-CoV-2, SARS-CoV and MERS-CoV are in ORF1a and the sequence of gene spike coding protein-S [5], which was identified as a key protein that interacts with target cells.

In terms of electron microscopic morphology, SARS-CoV-2 virions are generally spherical, but some are polygonal. The diameter is between 60 and 140 nm. The virus particles have prominent spines that are approximately 9 to 12 nm, which cause the virus to have a coronal shape. According to the virus morphology observed under the microscope, the virus is consistent with other in the coronavirus family, including SARS-CoV and MERS-CoV [5, 13].

The receptor on the target cells is the factor determining how the virus enters the cell and which tissues are susceptible, and the spike protein initiates the merging of the viral envelope with the host cell cytomembrane. Existing experimental studies have shown that ACE2 is likely to be the cell receptor of SARS-CoV-2, and SARS-CoV-2 does not use other coronavirus receptors. The main receptors of SARS-CoV and MERS-CoV are ACE2 and hDPP4 (human dipeptidyl peptidase 4 or CD26), respectively [1, 5, 14].

#### Clinical features

##### Symptoms

Although the study of COVID-19 is still in progress, our summary and comparison of coronaviruses can be useful for further research and clinical applications. The clinical symptoms of COVID-19 are similar to those of SARS and MERS, including fever, cough, myalgia and fatigue. Almost all of the patients have pneumonia, and their chest CT examinations are abnormal [1, 4, 15–17]. However, those who are infected with SARS-CoV-2 rarely have significant upper respiratory signs and symptoms, including nosebleed, sneezing or sore throat, which indicates that the target cell may exist in the lower respiratory tract. This is consistent with the autopsy reports of patients with COVID-19 that show that SARS-CoV-2 infection mainly causes deep airway inflammatory reactions and alveolar damage. Some patients may also have headache, hemoptysis, diarrhea, dyspnea and lymphocytopenia, but patients are less likely to have gastrointestinal symptoms [4]. Complications include acute respiratory distress syndrome, acute heart injury, and secondary infections. COVID-19 patients can be divided into those with asymptomatic, mild and severe cases. For most patients, the incubation period of the virus is generally 7–14 days. Typically, COVID-19 gradually progresses and worsens. Thus, each patient’s condition becomes more serious in the second week.

COVID-19, SARS, and MERS have different mortality rates. Among them, MERS had the highest fatality rate, and COVID-19 has the lowest fatality rate. It is worth noting that watery diarrhea is common in almost 60% of patients who suffer from SARS, and there is a typical biphasic clinical course [10, 18, 19]. In MERS, most patients have symptoms that include dry cough fever, malaise, myalgia, sore throat, headache, nausea, vomiting, and diarrhea, which are similar to the symptoms of SARS, but MERS has an unpredictable and erratic clinical course [19–22]. Fibrosis and consolidation in COVID-19 are less serious than the lesions caused by SARS, revealing that in COVID-19, the chest lesions are not primarily serous inflammation but rather are exudative reactions. Whether damage to the brain, myocardium, epicardium, kidneys, spleen and digestive organs is associated with viral infection needs further research.

##### Identification and diagnosis

Next-generation sequencing (NGS) and electron microscopy technology play critical roles in the early diagnosis of COVID-19, but their diagnostic values have been weakened by the use of specific nucleic acid detection technology [11, 23]. At present, clinically confirmed patients are usually diagnosed by collecting throat swabs and then detecting the nucleic acid of SARS-CoV-2.

Diagnosis based on clinical manifestations can be an early and rapid screening method. Patients with mild symptoms may not present positive signs. Patients in severe condition may have shortness of breath, moist rales in lungs, weakened breath sounds, dullness on percussion, and changes in voice, and the physical examination can help identify these symptoms. In addition, CT imaging plays an important role in the diagnosis. The imaging features of lesions show characteristic (1) distribution (mainly subpleural, along the bronchial vascular bundles); (2) quantity (often more than three lesions, occasionally single or double lesions); (3) shape (patchy, large block, nodular, lumpy, honeycomb-like or grid-like, cord-like, etc.); (4) density (mostly uneven, crazy-paving pattern mixed with ground glass opacity and interlobular septal thickening, consolidation and thickened bronchial wall, etc.); and (5) concomitant signs (e.g., air bronchogram, rare pleural effusion and mediastinal lymph node enlargement). However, these are not enough. COVID-19 needs to be distinguished from other known viruses that cause pneumonia, such as influenza virus, parainfluenza virus, adenovirus, respiratory syncytial virus, rhinovirus, human metapneumovirus, SARS-CoV, etc. and from Mycoplasma pneumonia, Chlamydia pneumonia, and bacterial pneumonia. In addition, COVID-19 should be distinguished from noninfectious diseases, such as vasculitis, dermatomyositis, and organizing pneumonia.

#### Treatments

##### Research on identifying effective drugs

Research on identifying effective drugs has started, and there have been many in vitro and in vivo experiments being conducted [24]. Vaccines against SARS-CoV-2 are currently in development, and there are at least two kinds currently ready for testing. There are approximately 15 potential vaccine candidates in the pipeline globally using a wide range of approaches (such as messenger RNA, DNA, nanoparticle, and synthetic and modified virus-like particles). The vaccine candidates will be developed by a number of organizations using DNA, recombinant and mRNA vaccine platforms109. On 23 January 2020, The Coalition for Epidemic Preparedness Innovations (CEPI) announced that they will fund vaccine development programmes with Inovio, The University of Queensland and Moderna, Inc., with the target of testing the experimental vaccines clinically. It will likely take approximately a year for most candidates to enter phase 1 clinical trials except for those funded by CEPI. For SARS, the vaccines in development include viral vector-based vaccines, DNA vaccines, subunit vaccines, virus-like particle (VLP)-based vaccines, inactivated whole-virus (IWV) vaccines and live attenuated vaccines, and the latest findings for these vaccines are based on the review by Yong et al. (2019) in August 2019 [25]. There was one SARS vaccine trial conducted by the US National Institute of Allergy and Infectious Diseases. Both Phase I clinical trials reported positive results, but only one will proceed to the Phase 2 trial. For MERS, there is only one published clinical study on a vaccine developed by GeneOne Life Science & Inovio Pharmaceuticals [26]. For therapeutics, there are nine clinical trials registered with the clinical trials registry (ClinicalTrials.gov) investigating therapeutic agents for COVID-19. Five studies on hydroxychloroquine, lopinavir plus ritonavir and arbidol, mesenchymal stem cells, traditional Chinese medicine and glucocorticoid therapy usage have commenced recruitment, and the other four are on antivirals, interferon atomization, darunavir and cobicistat, Arbidol, and remdesivir [24].

##### Chemotherapy schemes

COVID-19 patients admitted to a qualified hospital are given chemotherapy, including antiviral treatment, antibiotic therapy, corticosteroid therapy and other medications, such as ibuprofen as an antipyretic, nutrition support treatment, H_2_ receptor antagonists or proton pump inhibitors for gastrointestinal bleeding, and selective (M_1_, M_3_) receptor anticholinergic drugs for dyspnea, coughing, wheezing, and respiratory distress syndrome. Although α-interferon atomization inhalation and oral lopinavir/ritonavir can be considered, the effectiveness of the combined use of antivirals is still unknown, given the lack of evidence from a randomized controlled trial (RCT). Given the high risk of adverse effects, there are limitations on the use of corticosteroids. Traditional Chinese medicine has shown a good effect with regard to both prevention and treatment. Fumigating rooms with moxa and wearing perfumed Chinese herb bags can help prevent community transmission. Huoxiang Zhengqi capsules are recommended for hypodynamia accompanied by gastrointestinal upset caused by COVID-19. For hypodynamia and fever, Jinhua Qinggan granules, Lianhua Qingwen capsules, Shufeng Jiedu capsules and Fangfeng Tongsheng pills are recommended [23].

##### Nursing care

Nursing care is important for isolated and critically ill patients, as classified according to the guidelines. Isolated patients at home should monitor their body temperature and breathing regularly. Patients are given oxygen therapy via a nasal catheter or a mask, antiviral drugs, antibacterial drugs, symptomatic treatments, nutritional support and psychological counselling. Critically ill patients are monitored with regard to their vital signs, water-electrolyte balance, acid-base balance, and the functioning of various organs. In addition to nutritional support and psychological counselling, they need oxygen therapy and some special treatments. For example, if a patient develops moderate to severe ARDS, invasive mechanical ventilation with the patient in a prone position needs to be initiated [23, 27].

##### Clinical case report

According to Yang et al., the case fatality ratio (CFR) during the first weeks of the epidemic ranged from 0.15% (95% confidence interval [*CI*]: 0.12–0.18%) in mainland China excluding Hubei t 1.41% (95% *CI*: 1.38–1.45%) in Hubei Province excluding the city of Wuhan to 5.25% (95% *CI*: 4.98–5.51%) in Wuhan City based on data from the Wuhan Municipal Health Commission and the China and National Health Commission of China [28]. Chen et al. systematically described 99 cases of COVID-19 in Wuhan, China. Critically ill patients died of severe pneumonia, septic shock, respiratory failure and multiple organ failure (MOF). The authors reached a speculative conclusion that SARS-CoV-2 is more likely to infect older adult males with chronic comorbidities as a result of their weaker immune systems. In patients with severe coinfections, immune function is important in addition to the virulence of the pathogens. Old age, obesity, and the presence of comorbidities might be associated with increased mortality. In addition, a substantial decrease in the total number of lymphocytes indicates that SARS-CoV-2 consumes many immune cells and inhibits the body’s cellular immune function; therefore, a low absolute value of lymphocytes could be used as a reference index in the diagnosis of new SARS-CoV-2 infections in the clinic [29].

#### Epidemical features

It is essential to analyze the infection source, transmission route, susceptible population and replication rate, especially the intermediate host and the exact route of transmission, to find the best measures to prevent the further spread of COVID-19.

##### Infection source

The infection sources include patients, virus carriers, and infected animals that serve as viral reservoirs. Searching for the hosts of the virus, or for the infection sources, is a vital process in understanding the viral dynamics. SARS-CoV-2 has 96.2% genetic sequence similarity to the previously identified BatCoV RaTG13, suggesting that bats are most likely to be the host of SARS-CoV-2 [1, 3, 30, 31]. The cluster of cases in the seafood market was comprehensively analyzed, and sequence comparison revealed that pangolins are the most likely intermediate host for SARS-CoV-2 [30]. However, SARS-CoV and MERS-CoV were also identified as having zoonotic origins, and the animal reservoirs seemed to be bats [9, 32]. Although bat coronaviruses are genetically related, the intermediate hosts are involved in cross-species transmission, after which human-to-human transmission developed. In contrast to SARS-CoV-2, the intermediate host of SARS-CoV was mainly palm civets [9, 33, 34], and the intermediate host of MERS-CoV was thought to be dromedary camels [22, 35]. All three coronaviruses can be traced to bats, while there are different intermediate hosts involved in cross-species transmission. These three viruses have caused widespread epidemics that originated in animal reservoirs; the high morbidity and mortality levels have caused panic and substantial economic loss.

##### Transmission route

Viruses can directly infect people but can also infect one or more kinds of animals. Although these animals themselves do not cause disease, they can act as vectors for the virus and transmit it to humans; during this process, some viruses may mutate and evolve new characteristics. According to the experimental results of Peng et al. [5], SARS-CoV-2 can be transmitted through respiratory droplets and direct contact, confirming that while the main transmission route of SARS-CoV-2 is aerosols, other routes of transmission may exist. Moreover, a recent experiment conducted with recovering patients found that SARS-CoV-2 can also exist in the patient’s stool, suggesting that the fecal-oral route may be a route of transmisson [36]. Li et al. investigated cases of SARS and found that SARS was spread mainly by respiratory droplets [19]. By analyzing case data, Hui et al. also found that direct person-to-person transmission through close contact can also spread SARS-CoV [18]. MERS-CoV was mainly transmitted through close contact with infected family members or infected individuals in the hospital. Xiao et al. identified seven hypothesized transmission modes based on the three main transmission routes (long-range airborne, close contact, and fomite), and the infection risks associated with each hypothesis were estimated using the multiagent modeling framework. This showed that transmission occurred via both the long-range airborne and close contact routes [22]. Based on the available data, all three coronaviruses can be transmitted by breathing respiratory droplets that contain virions, which indicates that wearing masks is an effective means of protecting susceptible people. All three coronaviruses are transmitted from animals to humans and from humans to humans.

##### Susceptible population

There is no evidence that people with certain characteristics are not susceptible to COVID-19. The available data suggest that people of all ages who have close contact with patients can be infected by SARS-CoV-2 [36–38]. The general public is susceptible, and the data are still being updated daily. The elderly population and patients with basic diseases are more susceptible to severe illness after infection, and children and infants can also be infected by SARS-CoV-2 [39]. SARS-CoV had a tendency to affect healthier and younger persons, with a mean patient age of 39.9 years (range 1–91), and the male to female ratio was 1∶1.3, with a slight female predominance. MERS-CoV had a tendency to affect the elderly and frail populations, especially males, with a mean age of 56 years (range 14–94), and the male to female ratio was 3.3∶1 with a male predominance [8, 10, 40].

A commonly used measure of infectivity is the basic reproduction number (*R*_0_), which is the average number of people infected who pass the virus on to others without intervention. In other words, the value is equivalent to how many people can be infected by an average patient. The larger the *R*_0_ is, the harder it is to control the epidemic. Researchers have estimated the *R*_0_ to be in the range of 2.8–3.9, assuming extreme cases, which means that on average a COVID-19 patient passes the virus on to 2.8–3.9 healthy persons [28, 41]. In comparison, the *R*_0_ of MERS has been reported to be less than 1, and the *R*_0_ of SARS is estimated to be 3. Considering that the disease is now widespread around the world, the *R*_0_ of COVID-19 may change and could be higher than those of SARS and MERS.

##### Spreading speed

As of May 24, 2020, there were caused 84 536 confirmed cases of COVID-19, 4645 deaths and 79 757 cured cases in China. A total of 5 490 640 cases have been diagnosed, and 346 328 deaths have occurred worldwide. SARS infected more than 8098 people in 29 countries and caused 916 deaths, with a mortality rate of approximately 10%. MERS was first found in the Arabian Peninsula and infected approximately 2254 people (from 2012 through September 16, 2018) in 27 countries; MERS caused 800 deaths, with a mortality rate of approximately 35%. SARS was characterized by superspreading events, while COVID-19 is unique for its indiscriminate transmission among the general public. However, MERS seemed to be less aggressive [8, 10, 42].

#### Influences and measures taken in society

Epidemiological changes have been monitored, taking into account potential routes of transmission and subclinical infections. The official platform updates the public daily on the number of newly diagnosed cases, deaths and cures in each administrative region based on data from the Centers for Disease Control and Prevention and hospitals at all levels. Since the outbreak, many emergency measures have been taken to reduce person-to-person transmission of SARS-CoV-2. For example, public services and facilities provide disinfectants on a routine basis to encourage appropriate hand hygiene, and physical contact with wet and contaminated objects is considered when dealing with the virus, especially fecal and urine samples that can potentially serve as an alternative route of transmission. China and other countries have implemented major prevention and control measures, including screening travelers, to control further spread of the virus [43]. There are many people donating money, vegetables, medical supplies, etc. to the areas affected by the epidemic. In Wuhan, two hospitals, Vulcan Mountain Hospital and Raytheon Mountain Hospital, were built within 10 days, which can contain 1000 and 1300 patients, respectively. According to the People’s Daily, the National Health and Fitness Commission reported that there are more than 11 000 critical care workers and more than 2000 intensive care unit nurses, and there will be more pooling of medical resources in places where they are most needed. The Chinese government has shut down schools and closed businesses to reduce transmission [44].

The outbreak has also caused widespread public concern. Husnayain et al. studied the potential to use Google Trends (GT) to monitor public restlessness regarding the COVID-19 epidemic, and they found that searches related to COVID-19 and face masks increased rapidly [45]. With the advent of 5G and the rapid development of the information age, it may be more convenient for the masses to obtain the latest news from the Internet; thus, Internet-based risk communication is becoming an appropriate strategy. There are many disease control organizations and medical institutions that have played an official role in this outbreak and provided accurate and reliable information to the public in a timely manner. For example, laboratory confirmation of COVID-19 was performed in five different institutions, namely, the China CDC, Chinese Academy of Medical Science, Wuhan Institute of Virology, and Academy of Military Medical Sciences, and Chinese Academy of Sciences [29]. According to the CCTV news, with scientific progress has enabled the use of advanced technologies to control this epidemic. In addition, the health code divides the public into three health situations, namely, green, red and yellow. This provides an effective method of facilitating crowd tracking and monitoring. Furthermore, the geographic information system (GIS), which has long been used by many health professionals when tracking and combating contagion, also plays an important role in the geographical tracking and mapping of epidemics. A range of practical online/mobile GIS and mapping dashboards and applications have come into use for tracking the COVID-19 epidemic [46].

### Novel findings in clinical trials of treatments for COVID-19 and research priorities

#### Clinical trials for COVID-19

Some treatments have been adopted in clinical practice, and a few have been successful [24, 47]. According to Prashant Pradhan, the first case cured in seven days in the United States showed that the antiviral medication remdesivir may become one of the specific medicines for COVID-19; however, this remains to be verified through clinical trials [16]. According to the research by Wang, XF, et al. about the clinical manifestations and epidemiology in children with COVID-19 treated with lopinavir and ritonavir and without glucocorticoids and immunoglobulin, all 20 patients improved and were discharged from hospital. This may lead to the conclusion that children’s clinical symptoms of COVID-19 are nonspecific and milder than those in adults, which has significant clinical value [48].

#### Future research priorities

Future research priorities may be focused on biological research on SARS-CoV-2 and clinical research on COVID-19 diagnosis and treatment. According to Pradhan et al., there are four unique insertions, which have similarity to HIV, in the S-protein in COVID-19, which may explain its contagiousness. The gene binding site may become a new target of therapeutics to prevent transmission of the virus [49]. Specifically, virus particles are found in the feces, which suggests that there may exist other routes of transmission, such as fecal-oral transmission. Previously, we focused on cutting off transmission routes mainly by limiting contact and preventing respiratory droplet transmission. This finding emphasizes the significance of dealing with the feces of the patient. Therefore, for patients who already have COVID-19, careful disposal of their feces is an important concern with regard to reducing viral transmission [36]. On the basis of the research by Hongzhou Lu, lopinavir/ritonavir, nucleoside analogs, neuraminidase inhibitors, remdesivir, peptide (EK1), Arbidol, RNA synthesis inhibitors (such as TDF, 3TC), anti-inflammatory drugs (such as hormones and other molecules), Chinese traditional medicine and so on could be therapies for COVID-19, but the effects and safety remain to be tested in clinical trials [27].

### AutoDock simulation

#### Docking system test

**Fig. 2:**
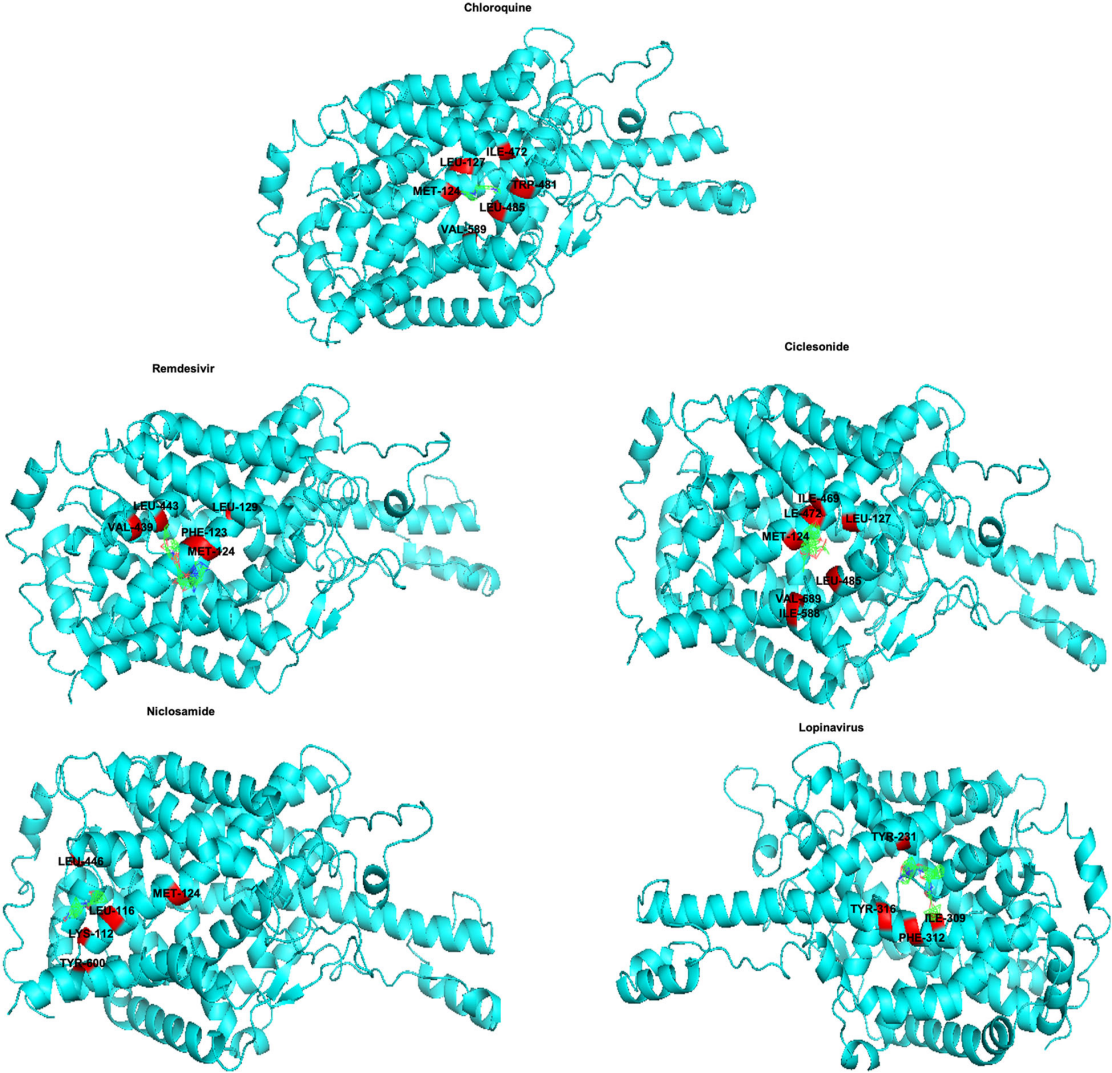
AutoDock calculations were performed to determine and compare the binding affinities of remdesivir, chloroquine, ciclesonide, niclosamide, and lopinavirus to ACE2. LEU: Leucine, PHE: Phenylalanine, MET: Methionine, VAL: Valine), ILE: Isoleucine, TRP: Ttryptophan, TYR: Tyrosine

3D structures of remdesivir, chloroquine, ciclesonide, niclosamide, and lopinavirus were obtained from NCBI PubChem. The crystal structure of ACE2 (PDB code: 6 M17) was obtained from the Protein Data Bank. The ligands within the crystal structure complex were extracted by PyMOL software (San Carlos, CA, USA). AutoDock 4.2 was used for the docking system test. AutoDock tools initialized the ligands by adding gasteiger charges, merging nonpolar hydrogen bonds, and setting rotatable bonds. The ligands were rewritten into PDBQT format, which can be read by Autodock software (AutoDock 4.2, San Carlos, CA, USA). AutoDock Tools were used to add polar hydrogen to the entire receptor. The grid box was set to contain the entire receptor region. The receptor output was also saved in PDBQT format. AutoDock Vina was set with the macromolecule held fixed and the ligands flexible. Affinity maps for all the atom types present, as well as an electrostatic map, were computed, with a grid spacing of 0.375 Å. The structural models were collected from the lowest-energy docking solution of each cluster of autodocks. It is evident from the findings of Fig. 2 and Table 2 that combinations of antiviral agents are more successful than a single drug.

**Table 2:** ACE2 binding sites and energies were evaluated through AutoDock calculations

ACE2	Amino Acid	Binding energy
Antiviral		
Remdesivir	LEU129, PHE123, MET124, VAL439, LEU443	-5.62
Chloroquine	MET124, LEU127, ILE472, TRP481, LEU485, VAL589	-4.2
Ciclesonide	MET124, LEU127, ILE472, LEU485, ILE469, ILE588, VAL589	-5.53
Niclosamide	LYS112, LEU116,MET124, LEU446, TYR600	-4.31
Lopinavirus	TYR231, ILE309, PHE312, TYR316	-5.5

*ACE2* Angiotensin converting enzyme 2, *LEU* Leucine, *PHE* Phenylalanine, *MET* Methionine, *VAL* Valine, *ILE* Isoleucine, *TRP* Tryptophan, *TYR* Tyrosine

#### Docking results and conclusion

The outbreak of SARS renewed interest in this family of viruses and resulted in the development of new drugs, among which remdesivir, chloroquine, ciclesonide, niclosamide, and lopinavirus are the most promising [50–52]. In addition, as mentioned above, ACE2 plays a vital role in the development of COVID-19 [53]. With regard to testing the effectiveness of previous medicines used by scientists for the treatment of diseases caused by coronaviruses, AutoDock calculations have been performed to classify specific binding amino acids and thus to determine the likely common cure targets for ACE2. As shown in Table 2 and Fig. 2, we found that chloroquine and ciclesonide share similar binding amino acid residues (MET124, LEU127, ILE472 and VAL589). Likewise, remdesivir and niclosamide also possess MET124. Taken together, we might therefore hypothesize that MET124 plays a key role in the efficiency of these drugs targeting ACE2. MET24 appears to be a potential target for COVID-19. However, there is no similar amino acid for lopinavir, suggesting that further studies are needed to elucidate the molecular mechanism of lopinavir treatment of COVID-19.

## Conclusions

The SARS-CoV-2 has a certain degree of homology with SARS-CoV and MERS-CoV. Compared to the previous SARS and MERS outbreaks, we found that COVID-19 has a few similarities with regard to the infection source and clinical symptoms, but it also some differences. For example, COVID-19 shares some routes of transmission in common with SARS and MERS, such as respiratory droplets, but COVID-19 can also be transmitted by the fecal-oral route [54–56]. It is important to consider all transmission routes when seeking to control the epidemic and protect medical staff and the general public. The epidemiological research on the source of infection and the route of transmission could help prevent the further spread of the epidemic. Different clinical symptoms could be used for the differential diagnosis of patients infected by coronaviruses. The medicines or therapeutic regimens that proved to be effective for SARS or MERS offer new approaches to the clinical treatment of COVID-19, which is essential in the prevention and control of the COVID-19 epidemic. The Chinese government’s control strategies in Wuhan and the active cooperation of the people have substantially contributed to China’s efforts to control the outbreak, which is important to the global efforts to combat the epidemic. Testing medications that have been shown to have antiviral effects against SARS-CoV or MERS-CoV may accelerate the pace of drug development in this emergency situation.

## Data Availability

Not applicable.

## Abbreviation

SsRNA: Single string RNA
ACE2: Angiotensin converting enzyme 2,
LEU: Leucine
PHE: Phenylalanine
MET: Methionine
VAL: Valine
ILE: isoleucine
TRP: Tryptophan
TYR: Tyrosine
